# Survivorship and growth in staghorn coral *(Acropora cervicornis)* outplanting projects in the Florida Keys National Marine Sanctuary

**DOI:** 10.1371/journal.pone.0231817

**Published:** 2020-05-06

**Authors:** Matthew Ware, Eliza N. Garfield, Ken Nedimyer, Jessica Levy, Les Kaufman, William Precht, R. Scott Winters, Steven L. Miller

**Affiliations:** 1 Department of Earth, Ocean and Atmospheric Science, Florida State University, Tallahassee, FL, United States of America; 2 Department of Biology, University of Hawaii at Manoa, Honolulu, HI, United States of America; 3 Reef Renewal, LLC, Tavernier, FL, United States of America; 4 Coral Restoration Foundation, Key Largo, FL, United States of America; 5 Marine Program and Pardee Center for the Study of the Longer-Range Future, Boston University, Boston, MA, United States of America; 6 Marine and Coastal Programs, Dial Cordy and Associates, Miami, FL, United States of America; 7 Halmos College of Natural Sciences and Oceanography, Nova Southeastern University, Dania Beach, FL, United States of America; College of William & Mary Virginia Institute of Marine Science, UNITED STATES

## Abstract

Significant population declines in *Acropora cervicornis* and *A*. *palmata* began in the 1970s and now exceed over 90%. The losses were caused by a combination of coral disease and bleaching, with possible contributions from other stressors, including pollution and predation. Reproduction in the wild by fragment regeneration and sexual recruitment is inadequate to offset population declines. Starting in 2007, the Coral Restoration Foundation^™^ evaluated the feasibility of outplanting *A*. *cervicornis* colonies to reefs in the Florida Keys to restore populations at sites where the species was previously abundant. Reported here are the results of 20 coral outplanting projects with each project defined as a cohort of colonies outplanted at the same time and location. Photogrammetric analysis and in situ monitoring (2007 to 2015) measured survivorship, growth, and condition of 2419 colonies. Survivorship was initially high but generally decreased after two years. Survivorship among projects based on colony counts ranged from 4% to 89% for seven cohorts monitored at least five years. Weibull survival models were used to estimate survivorship beyond the duration of the projects and ranged from approximately 0% to over 35% after five years and 0% to 10% after seven years. Growth rate averaged 10 cm/year during the first two years then plateaued in subsequent years. After four years, approximately one-third of surviving colonies were ≥ 50 cm in maximum diameter. Projects used three to sixteen different genotypes and significant differences did not occur in survivorship, condition, or growth. Restoration times for three reefs were calculated based on NOAA Recovery Plan (NRP) metrics (colony abundance and size) and the findings from projects reported here. Results support NRP conclusions that reducing stressors is required before significant population growth and recovery will occur. Until then, outplanting protects against local extinction and helps to maintain genetic diversity in the wild.

## Introduction

The tropical western Atlantic reef-building coral *Acropora cervicornis* was abundant and widespread throughout the Caribbean and Florida until the late 1970s [1]. The fast-growing coral formed dense thickets in forereef, backreef, and patch-reef environments to depths over 20 m [2–6] since the late Pleistocene [7, 8]. However, *A*. *cervicornis* abundance is now significantly reduced throughout its geographical range, primarily as a result of coral disease [1, 9, 10], bleaching [11–13], and other disturbances that affect sites at regional and local scales [14–16]. Today, *A*. *cervicornis* populations in Florida consist mostly of small (< 50 cm maximum diameter) and scattered colonies [17–19]. The dramatic decline in populations resulted in the loss of habitat complexity [20, 21], biological diversity [22], and the aesthetic quality of reef habitats. In the Caribbean, only a few populations survive that resemble the thickets of the past; these are in Honduras [23], the Dominican Republic [24, 25], and Belize [26, 27]. None persist in the Florida Keys, though a few nearshore aggregations exist north of Miami [28, 29] and in the Dry Tortugas [30].

Recovery has not occurred in Florida because stressors persist [31], including disease [9, 10], bleaching [14, 32], and episodic and severe cold-water events [33–38]. The mass mortality of the abundant grazing sea urchin *Diadema antillarum* in the early 1980s [39] also reshaped the ecology of coral reefs by releasing macroalgae from grazing [40]. There has since been a limited recovery of this herbivore in Florida [41, 42]. The dramatic decline in abundance and the absence of recovery of *A*. *cervicornis* and its congener *A*. *palmata* resulted in their listing as Critically Endangered on the IUCN Red List [43]. The corals are also listed as Threatened under the U.S. Endangered Species Act [44]. After the species were listed as Threatened in 2006 the National Oceanic and Atmospheric Administration (NOAA) developed a Recovery Plan for the two species [6].

The NOAA Recovery Plan (NRP) aims for population viability through increased population numbers and mitigation of stressors. Recovery criteria for the species include population metrics such as abundance, colony size, genetic diversity, and recruitment, that when met, along with the reduction of stressor impacts, would result in their delisting from the Endangered Species Act. The NRP identified coral outplanting of nursery-raised colonies to offshore reefs as a strategy to increase population numbers. The approach was based, in part, on a long history of projects that reattached corals dislodged by storms or by ship groundings [45–49], as well as outplanting advancements adapted from terrestrial silviculture practices and the aquarium trade [50–54]. These outplanting projects removed small pieces of coral from natural populations and propagated them by fragmentation to produce thousands of derivative colonies [55–58]. The colonies were subsequently outplanted in bulk to offshore coral reefs, where they were typically attached to the bottom using underwater cement or epoxy [55, 59, 60]. Colony numbers in individual nurseries quickly expanded from yearly production totals of hundreds to many thousands of colonies. As a result, restoration work advanced from restoring sites damaged by ship-groundings to projects that supplemented natural recruitment and enhanced existing populations [60–65].

While coral outplanting is considered a viable strategy to help meet the restoration criteria outlined in the NRP, coral propagation and outplanting are still a relatively new idea [66]. Though the practice is expanding rapidly and is now widely adopted by managers and restoration practitioners [67], few projects have been running long enough to assess their long-term potential to restore coral populations [48, 68–71]. An objective of the work reported here, based on results from longer-term *A*. *cervicornis* outplanting projects, is to determine the best approach to address and eventually overcome population declines. Results also inform the feasibility of success criteria identified in the NRP and help show how outplanted populations compare to baseline populations from the 1970s. Photographs and in situ field measurements documented how outplanting dates, site locations, habitat types, and genotypes relate to the survivorship, growth, and condition of nursery-raised *A*. *cervicornis* colonies outplanted to offshore reefs.

## Materials and methods

**Fig 1:**
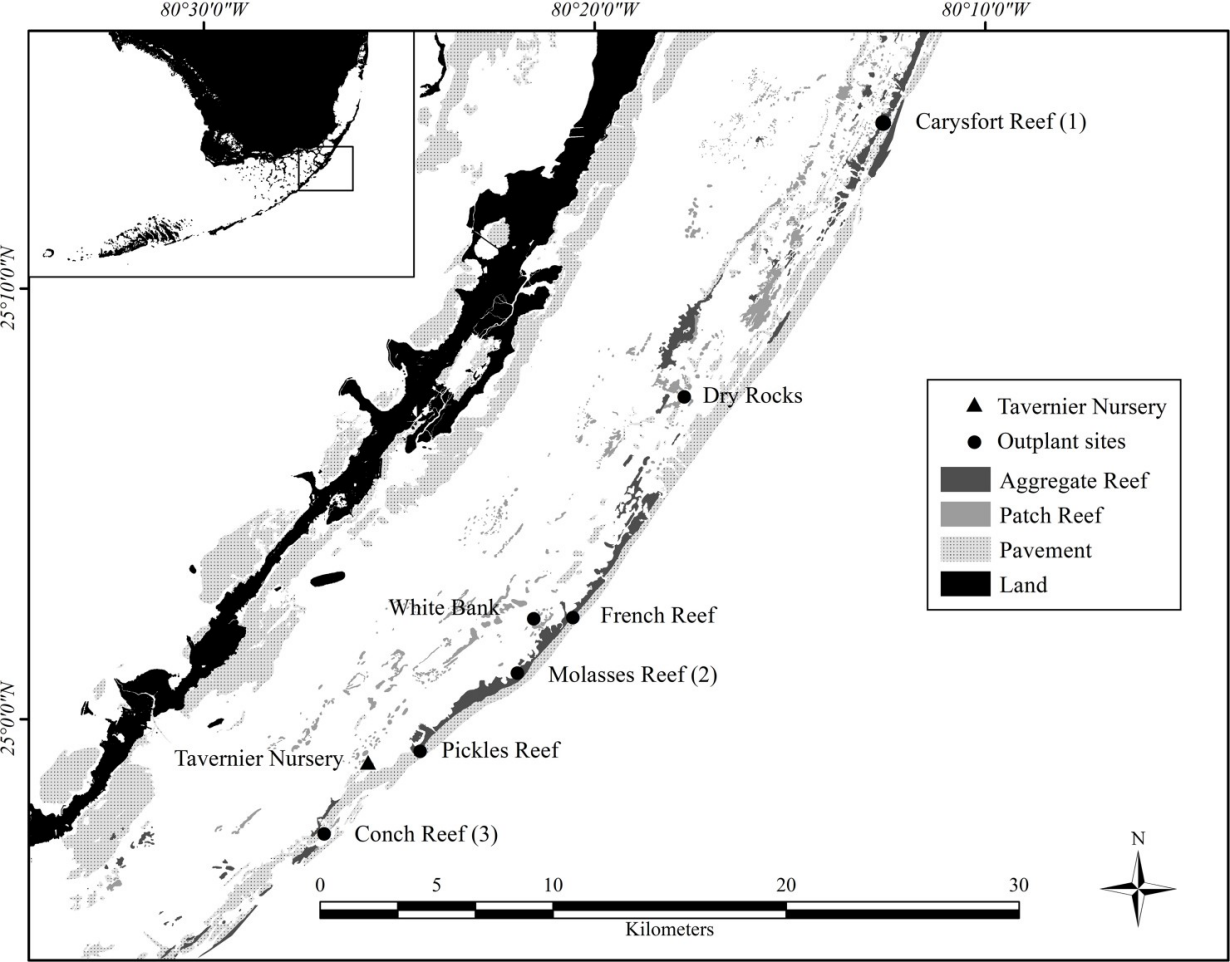
Location of the coral restoration foundation^™^ nursery (24.9882° N, 80.43633° W), coral outplant sites, and reef types. Reef types are from the Unified Florida Coral Reef Tract Map v2.0 (74). Carysfort (1), Molasses (2), and Conch Reefs (3) were used as examples to calculate restoration times based on NOAA Recovery Plan criteria.

The Coral Restoration Foundation^™^ (CRF) cultivated the *A*. *cervicornis* colonies used in this study in their coral nursery located 5 km offshore in the upper Florida Keys, over a sandy bottom, and at a depth of approximately 9 m (Fig 1). Coral fragments within the nursery were either fixed on disks or suspended from lines or tree structures [72, 73]. Colonies grew to approximately 15 cm maximum diameter before outplanting to reefs by CRF staff or by trained volunteers [55]. In 2013 larger colonies were also outplanted to Pickles (32 of 296), French (3 of 161) and Conch Reefs (1 of 92) that were approximately 30 cm maximum diameter, with a few larger than 40 cm. Twenty projects (Table 1), each defined by a cohort of colonies outplanted at the same time and location, were started between 2007 and 2013 at six different reefs in three benthic habitat types (Fig 1), including spur-and groove, patch-reef, and hardbottom [74]. The number of outplants per project ranged from 18 to 400, including three to 16 genotypes, increasing over time as nursery capacity increased and permits allowed. CRF maintains records-of-lineage for genotypes (identified by microsatellites and haplotype sequences of mt DNA) and colonies produced each year in the nursery.

**Table 1:** Coral outplanting project descriptions and results.

Project Site	Start Date	Habitat Type	Depth (m)	Config	Genotypes (#)	Colonies Outplanted	Survival #	Max Diameter (SE)	Duration (years)
**Molasses Reef**	2007 Jul	Spur & groove	7	T	3	18	2 (11%)	44.0 (26)	6.98
	2008 Oct	Hard-bottom	9	T	3	18	6 (33%)	47.0 (6.1)	5.76
	2009 Jan	Patch-reef	8	T	3	18	0	0	1.07
	2010 Jul	Patch-reef	8	T	3	24	0	0	0.95
	2012 May	Spur &Groove	7	O	10	400	164 (41%)	32.7 (1.5)	2.63
**Pickles Reef**	2008 Jul	Spur & groove	5	T	3	18	6 (33%)	35.7 (4.6)	6.28
	2009 Oct	Hard-bottom	9	T	3	24	1 (4%)	26.0 (0)	5.00
	2012 Apr	Hard-bottom	6	O	11	400	288 (72%)	28.9(1.0)	2.56
	2013 Jul	Spur & groove	7	C	16	296	207 (70%)	30.4 (1.1)	1.32
**White Bank**	2008 Aug	Patch-reef	10	T	3	18	0	0	1.57
**Dry Rocks**	2008 Nov	Spur & groove	9	T	3	18	16 (89%)	40.2 (5.1)	5.77
	2009 Jul	Spur & groove	5	T	3	24	16 (67%)	56.4 (6.1)	5.22
	2012 Jun	Spur & groove	8	O	11	400	190 (48%)	31.4 (0.9)	2.63
**French Reef**	2009 Apr	Spur & groove	9	T	3	18	14 (78%)	67.7 (6.0)	5.42
	2010 May	Spur & groove	9	T	3	24	2 (8%)	55.0 (19.0)	4.75
	2013 Aug	Spur & groove	10	C	10	161	104 (65%)	25.0 (1.5)	1.07
**Conch Reef**	2009 Aug	Hard-bottom	8	T	3	24	13 (54%)	NS	1.52
	2009 Oct	Hard-bottom	5	T	3	24	21 (88%)	NS	1.32
	2012 May	Hard-bottom	8	O	13	400	52 (13%)	26.7 (1.9)	2.67
	2013 Dec	Hard-bottom	5	C	10	92	82 (75%)	29.1 (1.5)	1.10

Outplant configurations and numbers (N) for the 20 projects included Triangle (T), Mixed Genotype Ovals (O) and Monogenetic Cluster (C). Duration is the time from initial outplanting to the last sampling date. NS is Not Sampled.

Outplanted colonies were attached to the reef with epoxy in three different configurations. Space between outplanted coral colonies allowed growth for one or two years before adjacent corals might touch [75]. This spacing within clusters reduced competition and disease transmission if colonies became infected. Early projects (2007–2010) positioned three colonies in a triangle approximately 1 m across with a different genotype at each corner. Later projects (2012–2013) placed colonies in 1 m diameter ovals or clusters, using a random arrangement of ten different genotypes for the ovals or monogenetic colonies for the clusters. No projects started in 2011.

Monitoring of outplanted corals included periodic visits to record general observations related to their condition (reflected by live tissue coverage), to attach broken fragments, and to photograph outplanted colonies. Re-attaching broken fragments increased initial outplanting success but quickly became impractical for projects that began with larger numbers of colonies. Photographs were used to follow each outplanting project for approximately two years. The photographs were matched to written project records to track outplant dates and locations, then screened for image quality, genotype tag identification, and scale information before a unique identifier code was assigned. Only projects with complete photographic records were analyzed. To be considered complete, a project included photographic documentation of all corals outplanted, with scale information, from at least two different visits. Survivorship, condition, and growth data were recorded in situ in 2014 and 2015 using SCUBA.

Twenty projects started between 2007 and 2013, and 2874 photographs (often with multiple colonies per photograph) and *in-situ* measurements provided a multi-year record of 2419 corals. Our analysis of individual cohorts was necessarily retrospective. The projects were not conducted as part of a larger experimental design to assess differences among start dates, habitat types, or reef sites. Also, projects included different sample sizes and monitoring dates. The 20 projects reported here comprise approximately one-third of the outplanting work conducted by CRF during this period. Not every CRF project was included in the analyses because, in addition to missing scale information or incomplete photographic documentation, permits required only a subset of total outplants to be monitored, so cohort analysis was not possible.

### Survivorship and condition

Survivorship (the percentage of colonies with any living tissue in a cohort) and condition (percentage of living tissue to the nearest 5%) [76] were obtained from the photographs using CANVAS software [77] and in situ using SCUBA. Weibull survival analysis models (using the statistical software package JMP, version 12 SAS) were used to project survivorship beyond the length of the studies [78]. Percent live tissue was analyzed in addition to survivorship because the survivorship metric is binary (dead = 0; alive = 1), while partial mortality is a continuous variable (0% to 100%) that impacts survivorship.

### Growth, size-frequency distributions, and genetics

The maximum skeletal diameter of colonies was measured using scale references in the photographs that included identifier tags (indicating genotype) of known sizes or PVC bars, or by direct measurements underwater using SCUBA. Growth was estimated using Gompertz growth functions: the general form is:

$$y=ae^{{-be}^{-ct}}$$

where y is the size, a is the maximum size asymptote, b is the displacement of the curve along the x-axis, c is the growth rate, and t is time. Due to gaps in the photographic record and reduced sample sizes, colonies that survived four years or longer were combined into a single group. Size measurements were not normally distributed, based on Wilks tests; therefore, log transformation was performed to better approximate a normal distribution. Statistical analyses using generalized linear models were performed using log-transformed data in R [79], including analyses to determine whether or not there were genotype effects on survivorship, condition and growth.

### GIS-based restoration analyses

The time (years) and effort (the number of outplanted colonies) required to restore Carysfort Reef, Molasses Reef, and Conch Reef (Fig 1) were estimated using results from this study and metrics in the NRP. Specifically, reef areas delineated by GIS were divided by survivorship estimates after four years for cohorts that started with 1050 colonies. Based on the NRP abundance and size metrics, each surviving colony that reaches ≥ 50 cm maximum diameter restores 1 m^2^ of the reef. Two depth ranges were used to calculate restoration areas for the three reefs: 5 to 10 m approximates the historical distribution of the species in the Caribbean and Florida [2, 80] and 5 to 20 m water depth as identified in the NRP. The three reefs are management zones in the Florida Keys National Marine Sanctuary, with boundaries that constrained the area estimates. Areas were delineated in GIS using a two-step geoprocessing intersect procedure. First, the Florida Keys 100 m grid cell habitat layer [17, 81] was clipped using the FKNMS management-zone layer. Then, the resulting habitats-within-zones layer was overlaid with the South Florida water depth layer. The final clipped and intersected layer contained polygons annotated with zone, habitat, and depth information.

### Permits

The following permits supported work in the Florida Keys National Marine Sanctuary: FKNMS-2008-006, FKNMS-2008-006-A2, FKNMS-2008-025, FKNMS-2008-026, FKNMS-2008-053, FKNMS-2009-099, FKNMS-2010-103, FKNMS-2011-150-A1, FKNMS-2011-159 (A1, A2, A3, and A4), SAL-13-1086-SCRP. All necessary permits were obtained for the described study, which complied with all relevant regulations.

## Results

### Survival and condition

Survivorship results based on individual colony counts for each cohort at the last monitoring date are presented in Table 1. Without an experimental design to test whether or not the start dates, reef sites, or habitat types affect survivorship, we instead used a retrospective approach to evaluate project results. Individual cohorts exhibited significant variability in survivorship. Seven of 20 cohorts had colonies that survived over five years, ranging from a low of 4% to a high of 89%. The two cohorts monitored the longest started with 18 colonies outplanted in 2007 and 2008, with 11% and 33% survivorship after nearly seven and just over six years, respectively. Five of the 20 cohorts had survivorship of 11% or less, ranging from one to five years in duration. The four projects with the largest cohorts to start (400 colonies) were monitored less than three years because they all began in 2012 or 2013. Survivorship results from these projects ranged from 23% to 72%.

**Fig 2:**
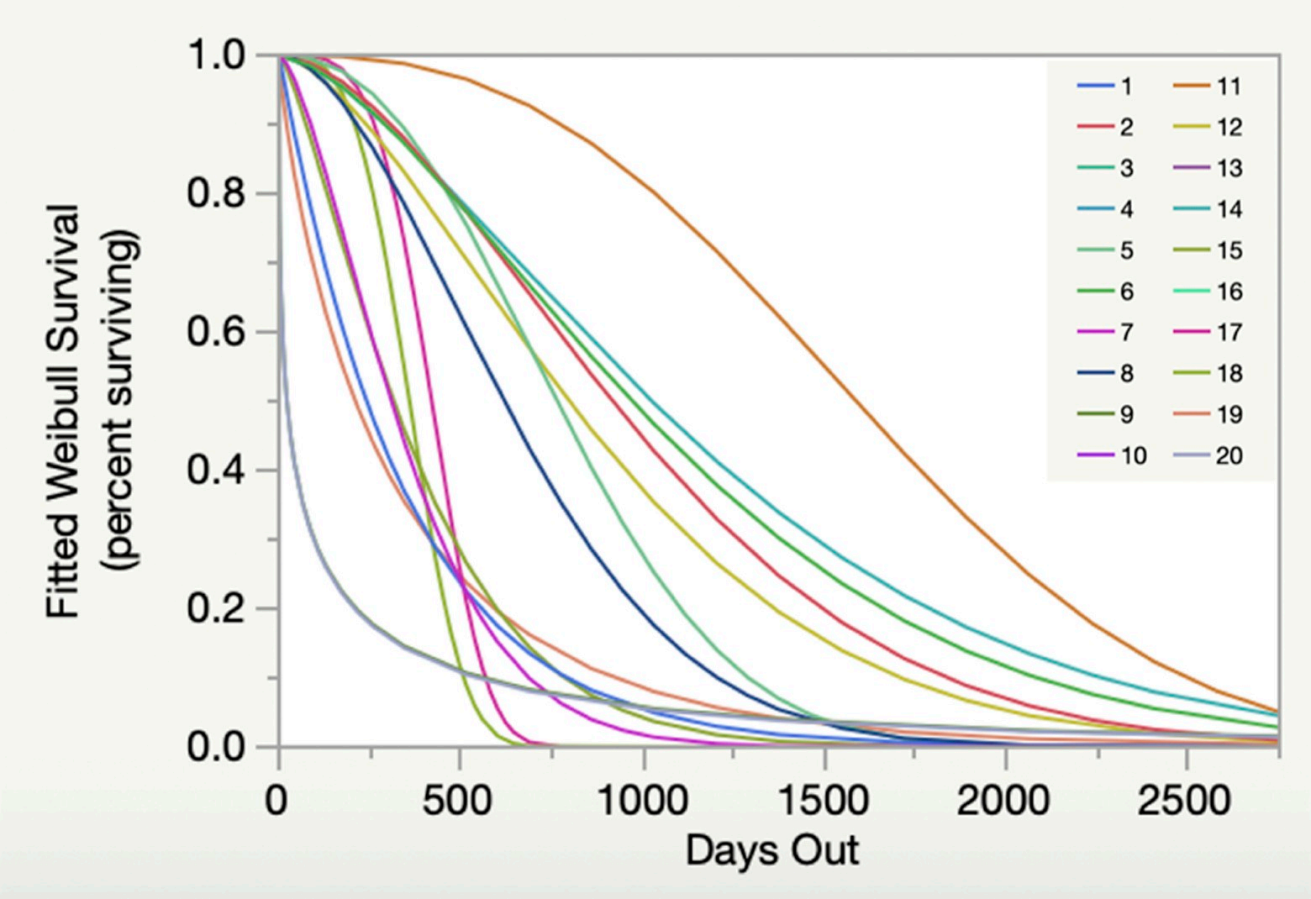
Weibull survival among cohorts. Individual cohorts are identified by numbers as follows: Molasses Reef (1–5; 2007, 2008, 2009, 2010, 2011), Pickles Reef (6–9; 2008, 2009, 2012, 2013), White Bank (10; 2008), Dry Rocks (11–13; 2008, 2009, 2012), French Reef (14–16; 2009, 2010, 2013), Conch Reef (17–20; 2009a, 2009b, 2012, 2013). Cohort details are presented in Table 1.

**Fig 3:**
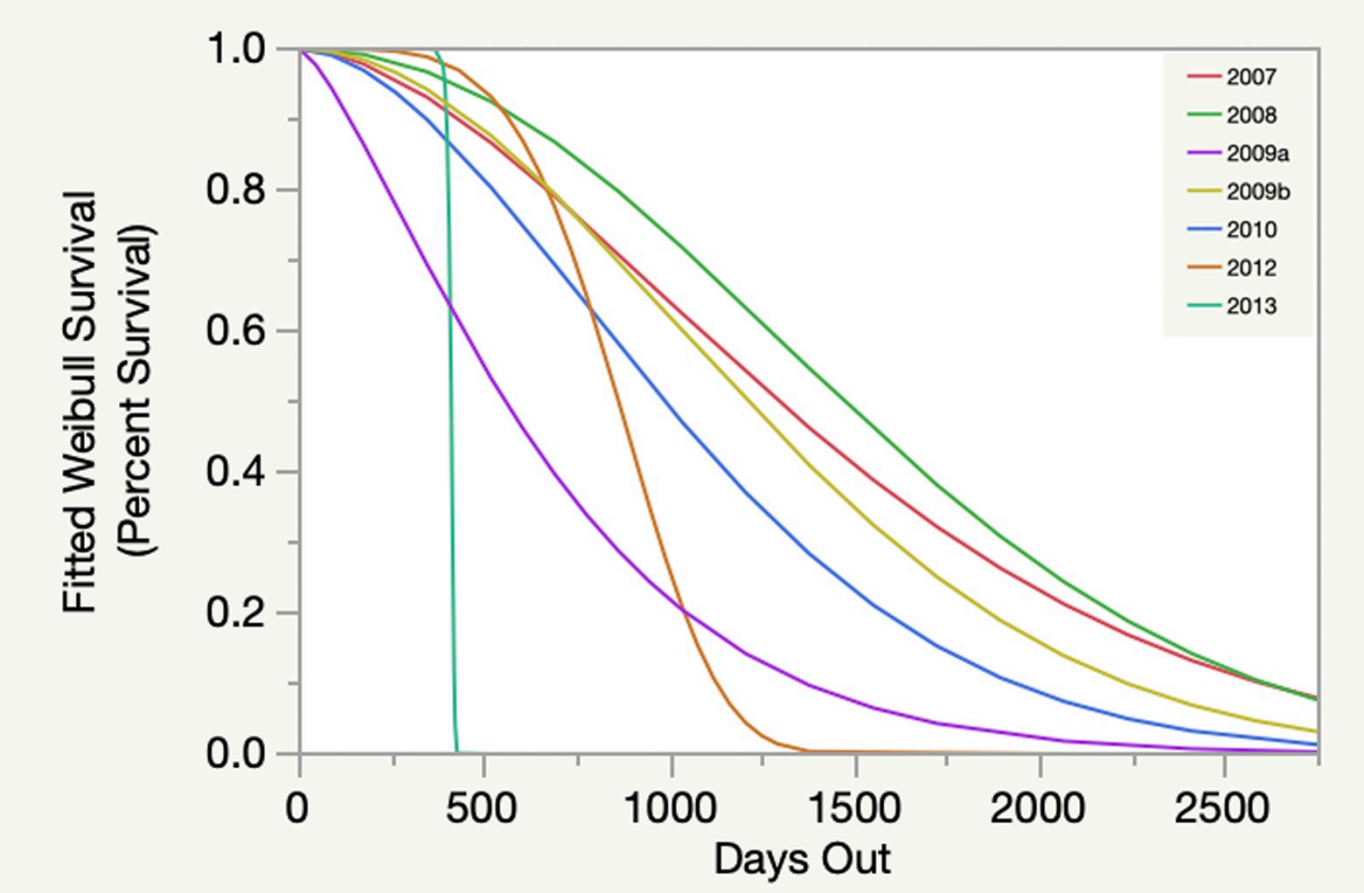
Weibull survival over time by year outplanted. Different years are indicated by different colors. 2009a and 2009b distinguish outplanting projects started in winter/spring versus summer/autumn, respectively.

**Fig 4:**
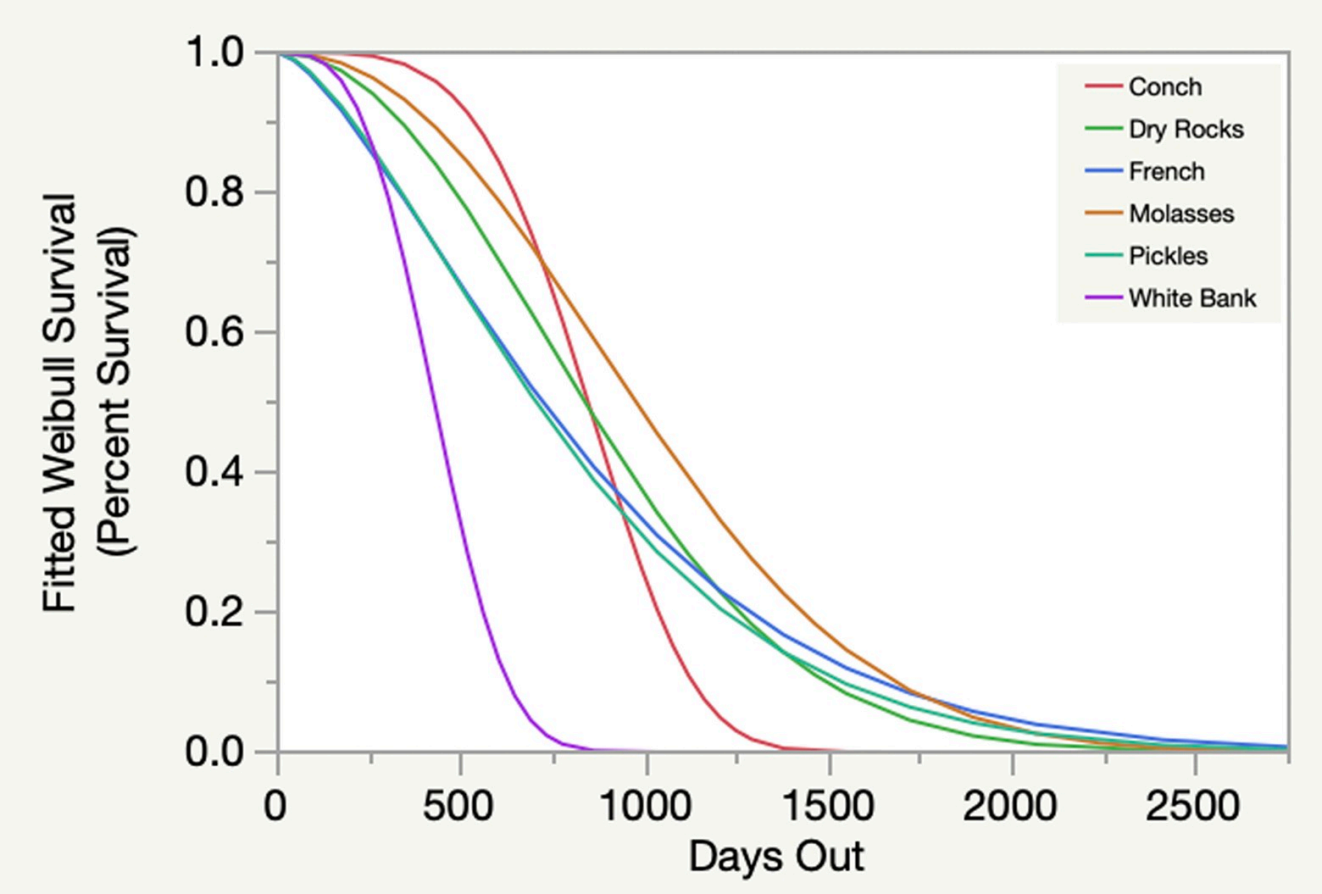
Weibull survival over time by reef location. Different reef locations are indicated by different colors.

**Fig 5:**
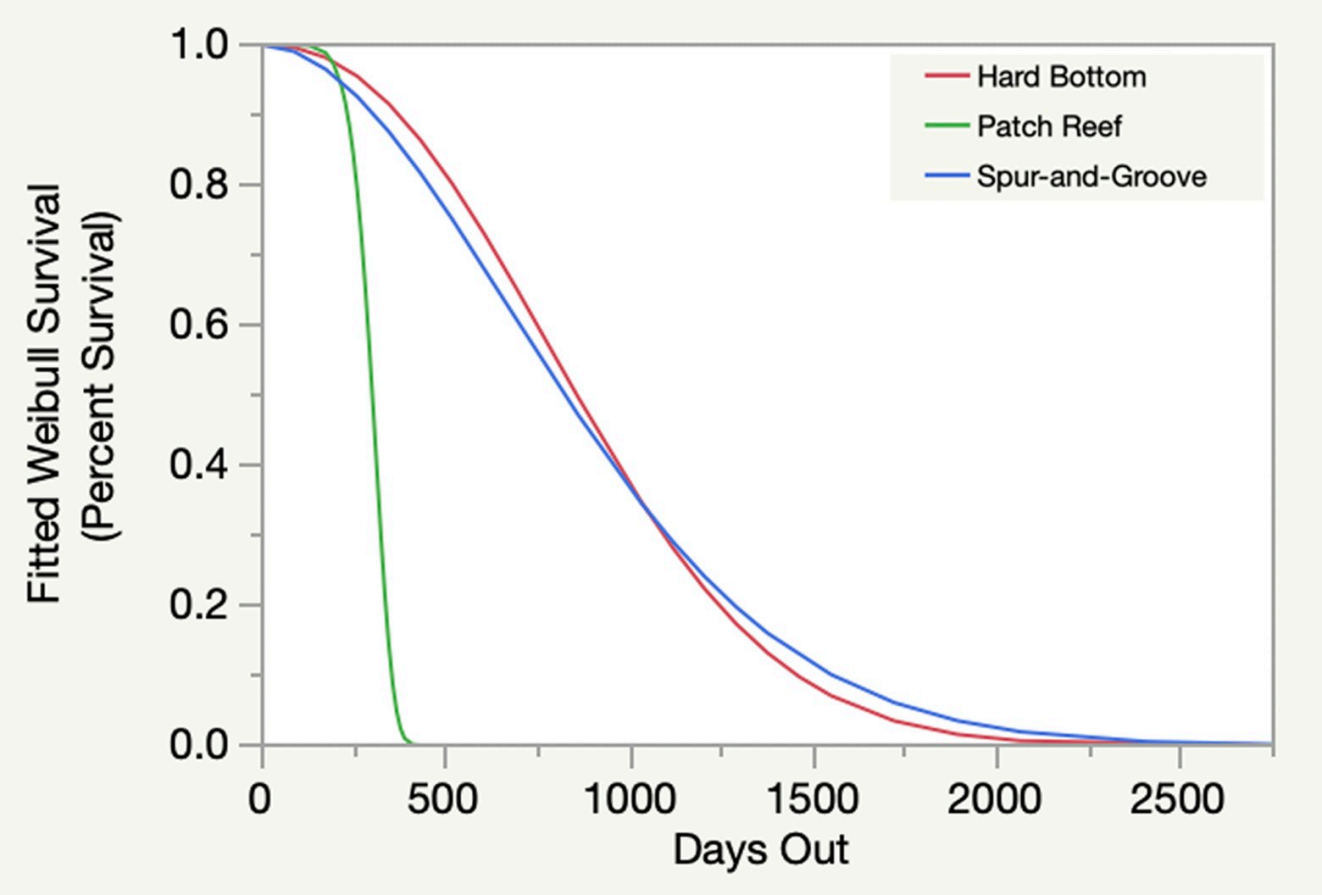
Weibull survivorship over time by habitat type. Different habitat types are indicated by different colors.

Weibull survival analysis also revealed significant variability among individual cohorts (Fig 2). A clear pattern of decline was evident over time, with survivorship between 0% and approximately 10% after seven years. Survivorship at five years ranged from approximately 0% to 35%. Similar results were seen when analyzed by start date (Fig 3), but when analyzed by reef site (Fig 4) and habitat type (Fig 5) survivorship at five years was approximately 10% or less.

The condition of outplanted colonies (Table 2) based on percent live tissue measurements remained greater than 85% the first two years, corresponding with similar high colony survivorship over the same period. After four years, the condition of surviving colonies declined to 50%. There were no significant differences among cohorts after four years. Genotype effects related to survival (Kruskal-Wallis p = 0.49) and condition (Kruskal-Wallis p = 0.14) were not statistically significant. It is important to note that the earliest projects comprised smaller sample sizes for outplanted colonies and fewer genotypes (Table 1).

**Table 2:** Percent live tissue coverage (i.e. condition) of outplanted colonies over time.

Condition	Year 1	Year 2	Year 3	Year 4
Mean % (± SE)	85.2 (1.1)	91.7 (1.8)	60.5 (1.6)	50.3 (4.0)
N	724	197	769	132

The apparent increase in live tissue coverage at Year 2 is likely due to the reduced sample size (a result of fewer suitable photographs that were available to analyze.)

### Growth, size-frequency distributions, and genetics

**Fig 6:**
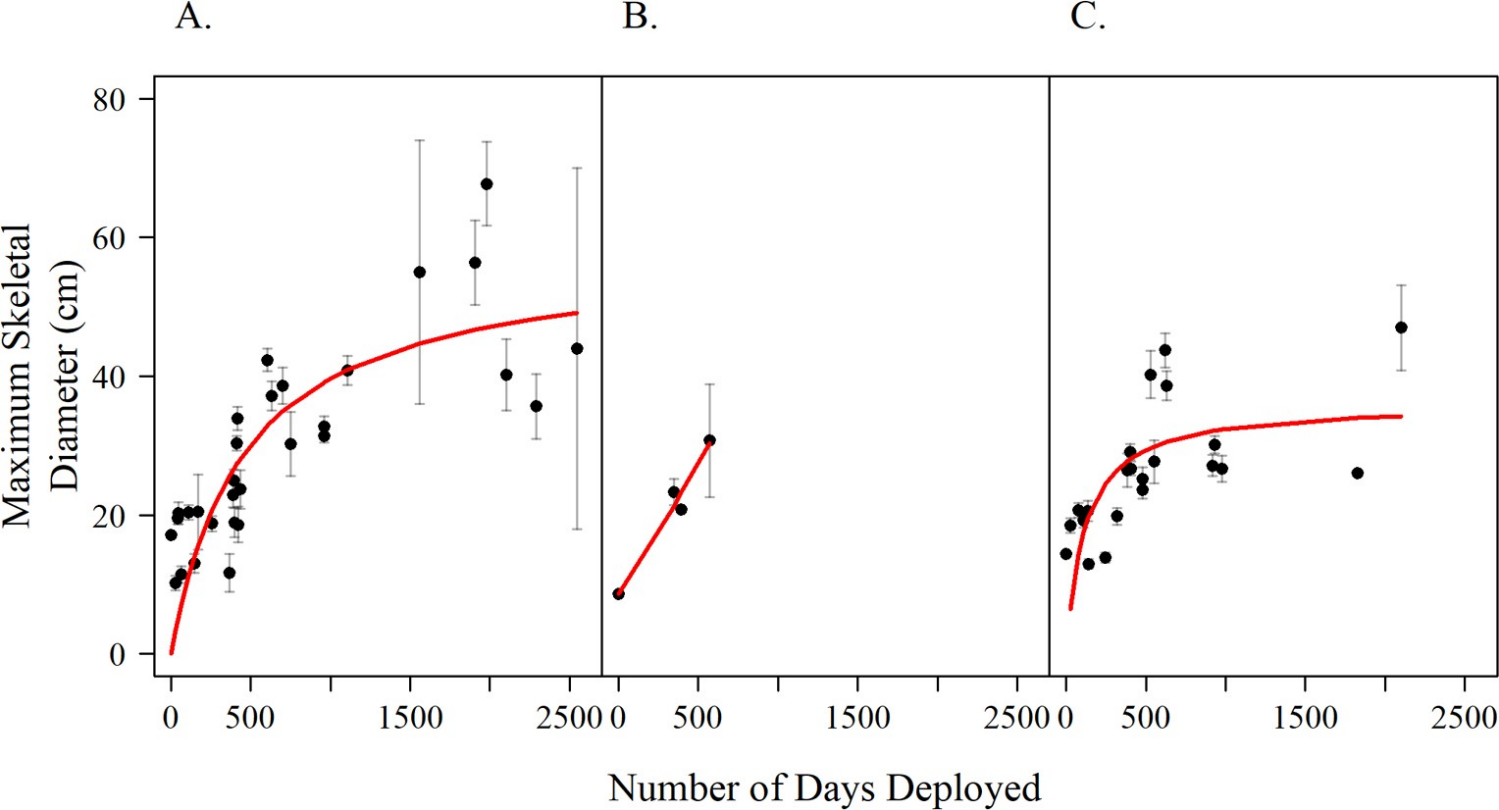
Maximum skeletal diameter of *A*. *cervicornis* colonies over time by habitat type. A) spur-and-groove, B) patch-reef, and C) hardbottom. Dots represent sample dates. Error bars represent standard error. Trendlines are Gompertz growth functions.

Colonies in spur-and-groove habitats (Fig 6) had a larger mean colony diameter (37.5 cm ± 1.4 SE) after two years than those on hard-bottom sites (31.9 cm ± 1.4 SE, pairwise Wilcoxon p = 0.014). Mean colony diameter did not differ significantly between patch-reef sites (30.7 cm ± 8.1 SE) and hard-bottom sites or between patch-reef sites and spur-and-groove sites (pairwise Wilcoxon, p = 1.0 for both comparisons). However, the patch-reef colonies suffered significant mortality during the first year and after, so their growth records are shorter. The maximum average size for colonies in spur-and-groove habitats was 48.8 cm (± 3.3 SE), which was not significantly different from that in the hard-bottom site colonies (44.0 cm ± 6.0 SE, pairwise Wilcoxon p = 0.85). The Gompertz growth functions for each habitat type, are as follows:

$${Spur‐and\;Groove\;y}_{spur}=50.88e^{{-1.363e}^{-0.0017t}}$$

$$Patch\;Reef\;y_{patch}=69.46e^{{-2.066e}^{-0.0016t}}$$

$$Hard‐bottom\;y_{hard}=35.40e^{{-0.926e}^{-0.0030t}}$$

where *y* equals maximum skeletal diameter (cm) and *t* is the number of days at-large.

**Fig 7:**
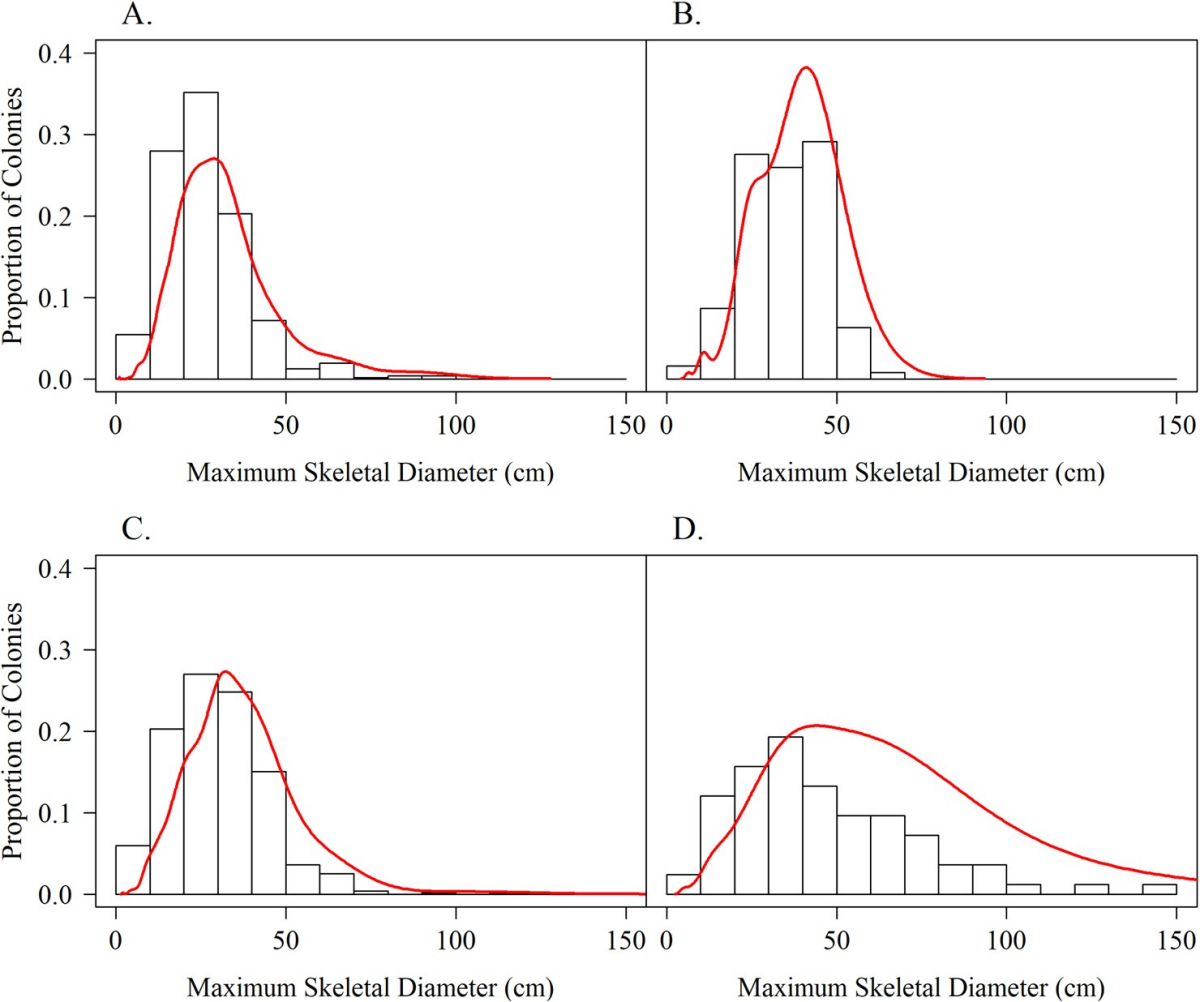
Size-frequency distributions for outplanted *A*. *cervicornis* colonies by project duration. A) Year 1 (N = 724); B) Year 2 (N = 197); C) Year 3 (N = 769); and D) Year 4+ (N = 132) (outplanted colonies combined from all projects that survived four years or longer). Curves are based on log transformations. The largest colony sizes in Year 1 are from the 2013 cohorts that included 42 colonies (out of 542 total) with larger initial maximum diameters of 30 cm.

The size-frequency distribution of colony sizes by outplant duration are presented in Fig 7. The largest colony sizes in Year 1 are from the 2013 cohorts that included 42 colonies (out of 542 total) with larger initial maximum diameters of 30 cm. It is also possible that fusion occurred among some colonies after one year. Results on both the arithmetic scale and the log-transformed data indicate a shift in the curve to the right and a flattening through time. Means and modes generally increased over time (Table 3), except in year three, which likely reflects large differences in sample sizes among years and different mortality rates among different cohorts. After four years, > 37% of the surviving outplants were larger than 50 cm in diameter, with 16.5% of colonies larger than 100 cm. The mode of colony diameter for each year class also generally increased over time, reaching 52.3 cm after four years. Kurtosis decreased through time indicating movement toward the normal distribution, as colonies from the nursery were initially approximately the same size. The positive skew in all four years indicates that the outplant population tended to be dominated by smaller colonies. Despite changes in mean colony size, the coefficient of variation was largely unchanged through time indicating that variability increased with larger mean colony size.

**Table 3:** Statistics based on size-frequency distributions of outplanted *A*. *cervicornis* populations.

Metric	Year 1	Year 2	Year 3	Year 4
**Mean (SE) cm**	26.1 (0.5)	34.7 (1.0)	30.4 (0.6)	48.4 (3.0)
**Mode (cm)**	31	34.7	30	52.3
**CV**	0.5	0.3	0.5	0.6
**Skewness**	1.5	-0.1	0.9	1
**Kurtosis**	4.1	-0.6	2.35	1.3
**N**	724	197	769	132

N is the combined number of coral colonies in all projects at each time interval.

Genotype identification during in situ monitoring visits was not always possible due to identifier-tag losses. Genotypes in different habitat types did not exhibit statistically significant differences in percent-survival or condition (Kruskal-Wallis p = 0.48 and p = 0.14, respectively). No significant difference in colony diameter was evident based on results for 18 genotypes (Kruskal-Wallis p = 0.13), though a couple of pairwise Wilcoxon growth comparisons were suggestive at p = 0.058 and p = 0.56).

### GIS-based restoration analyses

**Fig 8:**
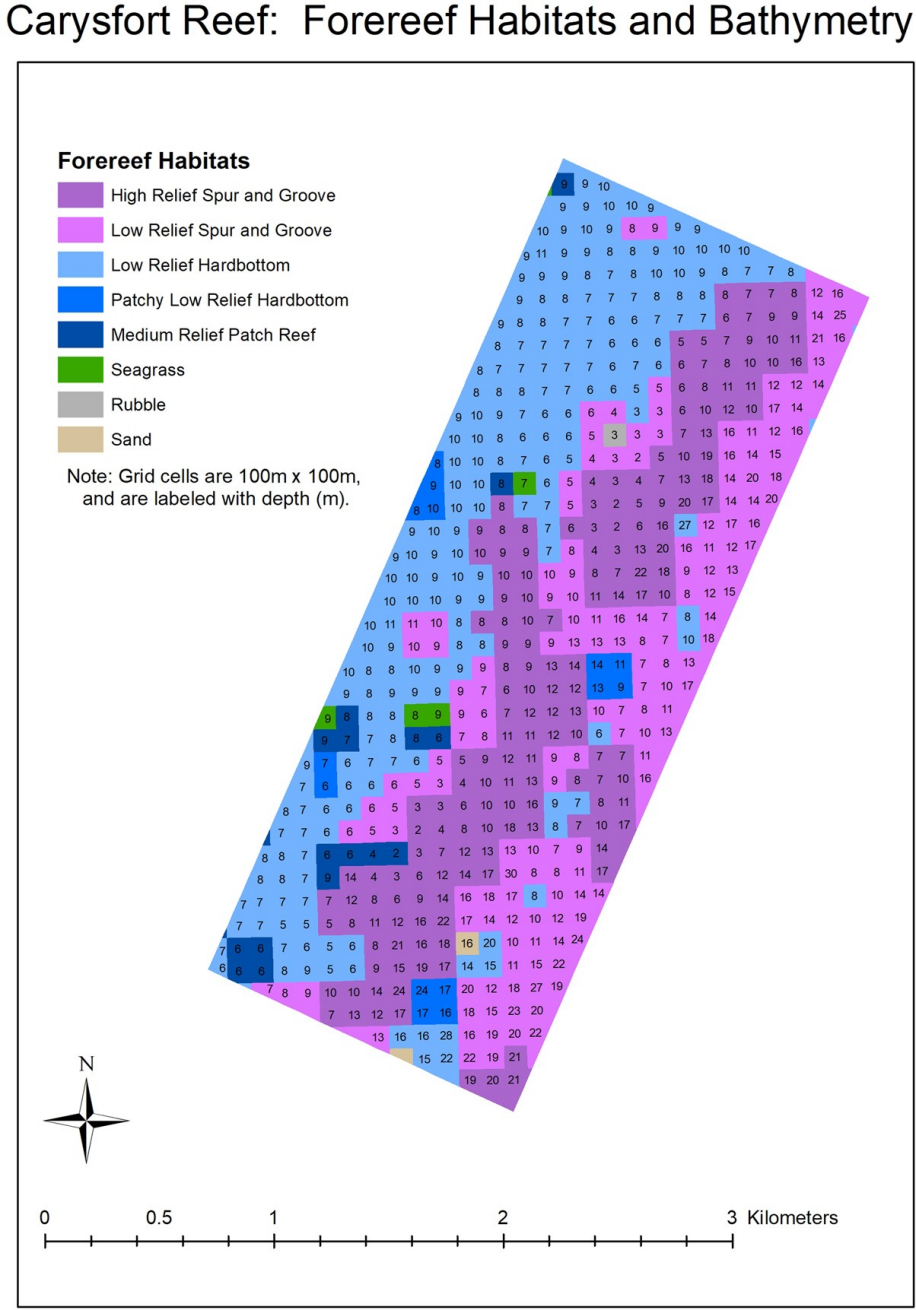
Forereef habitats and bathymetry for the carysfort reef sanctuary preservation area, located in the Florida Keys National Marine Sanctuary.

**Fig 9:**
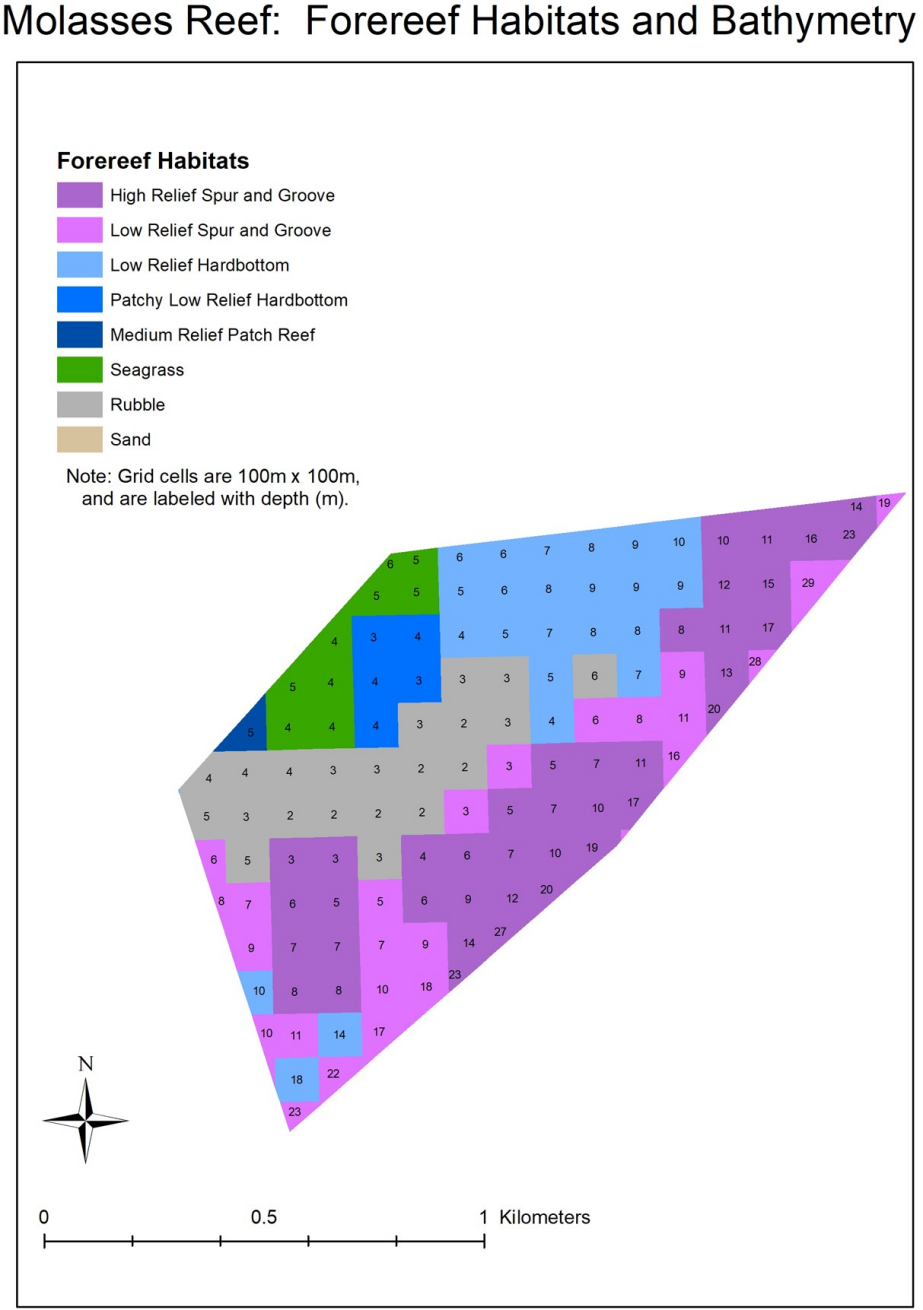
Forereef habitats and bathymetry for the molasses reef sanctuary preservation area, located in the Florida Keys National Marine Sanctuary.

**Fig 10:**
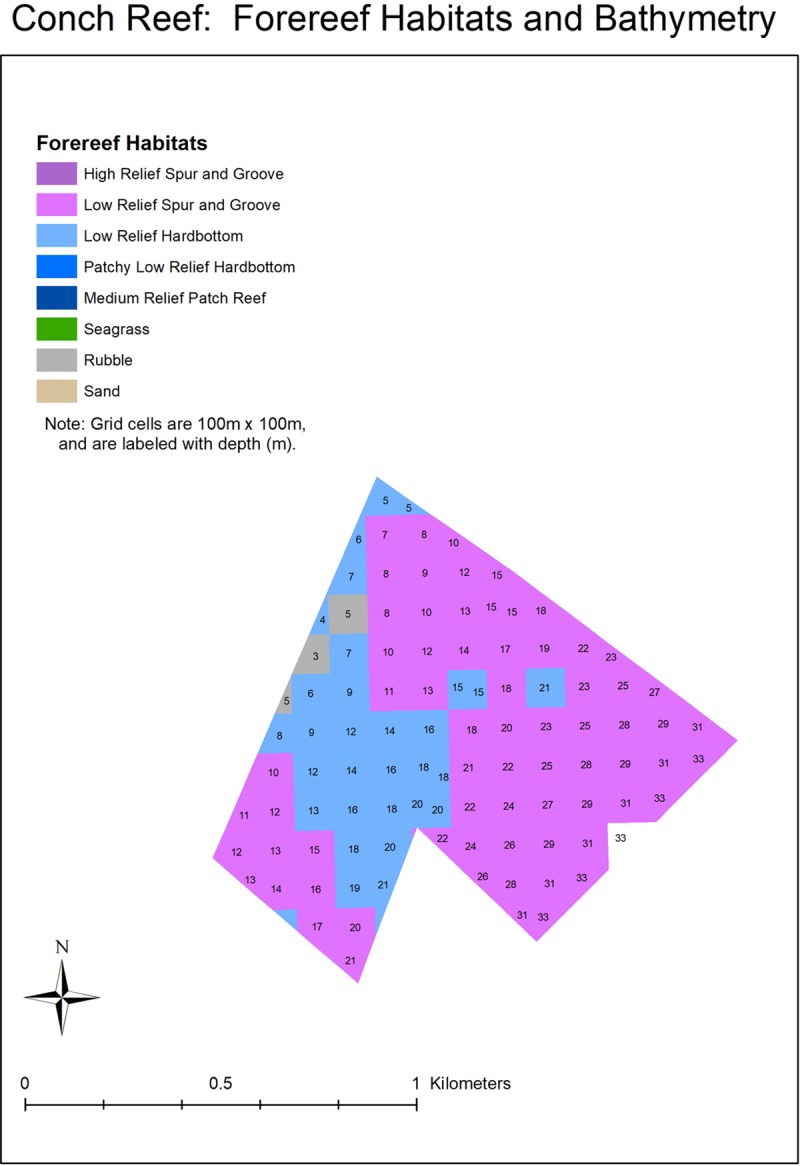
Forereef habitats and bathymetry for the conch reef sanctuary preservation area, located in the Florida Keys National Marine Sanctuary.

Results based on the fitted Weibull curves (Figs 2–5) suggest survivorship of approximately 15% to 40% after four years. This range of survivorship is similar to colony counts for the cohorts (Table 1). Colony growth after four years averaged nearly 50 cm maximum diameter (Fig 6) with approximately one-third of surviving colonies after four years this size (Fig 7). Therefore, starting with a cohort of 1050 colonies and assuming survival rates between 15% and 40%, between 158 and 420 colonies survive to four years post-outplanting and between 52 and 139 colonies of these reach the ≥ 50 cm maximum diameter size criterion specified in the NRP. Using the abundance criterion (1 colony/m^2^) in the NRP, rough estimates of the total restored area that results from 1050 colonies after four years is between 52 m^2^ (1050 outplanted colonies x 15% survivorship x 33% of surviving colonies ≥ 50 cm maximum diameter) and 139 m^2^ (1050 outplanted colonies x 40% survivorship x 33% of surviving colonies ≥ 50 cm maximum diameter). Based on the GIS-derived habitat areas (S1 Table) for Carysfort Reef (Fig 8), Molasses Reef (Fig 9) and Conch Reef (Fig 10) in the upper Florida Keys (Fig 1), restoration times using the two survivorship estimates of 15% and 40% are presented in Table 4. NOAA identified the 5% coverage criterion to represent “a small portion of potential core habitat strata with the assumption that, under this condition, additional lower-density stands would occupy additional habitat strata.” We use the 5% criterion to model these three reefs, which should be distinguished from the 5% NOAA criterion that was designed to apply across the entire region.

**Table 4:** Reef areas and restoration efforts (years) for three reefs in the Florida Keys.

GIS-derived habitat areas	Carysfort Reef (m^2^)	Molasses Reef (m^2^)	Conch Reef (m^2^)
Total reef area (5–20 m)	4,913,499	581,121	518,707
5% (5–20 m)	245,675	29,056	25,935
5% (5–10 m)	166,876	19,393	6,679
**Restoration Time (years)**	**15% Survivorship**	**15% Survivorship**	**15% Survivorship**
Total reef area (5–20 m)	94,490	12,298	10,978
5% (5–20 m)	4,725	615	549
5% (5–10 m)	3,209	410	141
**Restoration Time (years)**	**40% Survivorship**	**40% Survivorship**	**40% Survivorship**
Total reef area (5–20 m)	35,349	4,181	3,732
5% (5–20 m)	1,768	209	187
5% (5–10 m)	1,201	140	48

Reef areas for total area and two depth ranges that were used to calculate restoration efforts based on 15% and 40% survivorship after four years. GIS-derived habitat areas are from S1 Table.

## Discussion

The 2006 listing of *Acropora cervicornis* and *A*. *palmata* as Threatened under the U.S. Endangered Species Act raised awareness and helped to increase funding to support coral restoration in Florida and the Caribbean. Initial project results documented high survivorship for outplanted corals but few studies were monitored beyond a year or two [75, 82]. Similarly, survivorship in the present study was generally high during the first two years but mortality increased after this point with substantial variability among cohorts. The primary goal of monitoring conducted through 2010 was to evaluate the feasibility of outplanting to increase population numbers on offshore reefs. While the number of outplanted corals was small in these early projects, for cohorts monitored at least five years survivorship ranged from 0% to 89% (Table 1). The highest survivorship was for a 2008 cohort outplanted in spur-and-groove habitat at Dry Rocks. The results suggest that cohorts at Dry Rocks did well, with 67% survivorship after 5.2 years for a cohort started in 2009. However, more work is needed because sample sizes were small in terms of outplanted colonies and the number of cohorts monitored. For example, a 2009 cohort with 18 colonies outplanted at French Reef had 78% survivorship after 5.4 years, but a second cohort of 24 colonies outplanted in 2010 had 8% survivorship after 4.8 years. The former cohort was not affected by the 2010 cold-water event [36–38], which suggests that while there may be site-specific differences, episodic events can also be variable in their impact. In addition, the lack of a formal experimental design did not allow us to identify variables that might predispose cohorts to have greater survivorship based on start dates, reefs sites, or habitat types. After 2010, permit restrictions were relaxed and the number of corals outplanted increased more than fifteen-fold. Longer-term results from these projects are only available for several years because monitoring did not occur after 2015.

The Weibull models (Figs 2–5) are useful because they provide survivorship projections beyond the time frame of the study, but with caveats. Typically, the shape of the curves and confidence intervals can be used to compare different populations and to estimate metrics that determine whether or not the risk of failure (death) increases or decreases through time. However, these additional metrics are not appropriate because of the variable start dates and sample sizes. Instead, the curves allow visualization to estimate short- and long-term survivorship. When analyzed by cohort (Fig 2), Weibull survivorship models had similarly high variation compared to colony counts for individual cohorts (Table 1). However, the Weibull curves beyond seven years estimate survivorship less than 10 percent. Therefore, significant challenges remain to achieve high long-term survivorship routinely. Weibull modeling exhibited less variability for cohorts by start date (Fig 3), followed by reef sites (Fig 4), and habitat types (Fig 5), which resulted from pooling cohort results by treatments. Survivorship at seven years by start date was similar to what was seen for the cohort analyses, but approached 0% for the Weibull curves by reef sites and habitat types. Reef site variability (Fig 4) was shaped on the low end by White Banks that comprised one patch reef cohort with 100% mortality within the first two years, caused by the 2010 cold-water event. Two Molasses Reef cohorts died the first year, and the largest cohort of 400 outplants had 41% survivorship after 2.6 years. For habitat types (Fig 5), patch reefs comprised only two cohorts, both of which died in the first year. For spur-and-groove and hardbottom habitats (Fig 5), the sample sizes included ten and seven cohorts, respectively. Survivorship results ranged from 11% to 78% for spur-and-groove habitat and 4% to 88% for hardbottom habitat. While the Weibull models for spur-and-groove and hardbottom habitats appear nearly identical, it is premature to assume that habitat type does not impact survivorship without explicitly testing for this result.

Colony growth results for the first two years documented rates that are similar to many historical populations of *A*. *cervicornis* [83, 84] and other coral outplanting projects [85, 86]. After two years, colony growth generally slowed. However, approximately 15% of outplanted colonies that survived four years attained greater than 100 cm maximum colony diameter, so at least a small proportion of outplanted colonies retain the capacity to grow as fast and large as the largest colonies in natural local populations [18]. Larger maximum colony diameter was achieved after two years in spur-and-groove habitat compared to hard-bottom (Fig 6) which may reflect greater hydrodynamic flow in the former that results from increased topographic complexity [87–90]. Maximum colony diameter did not always correlate with survivorship or habitat type. For example, survivorship after three years at Dry Rocks (spur-and-groove) was nearly two times higher than at Conch Reef (hardbottom) for 2012 cohorts (Table 1) but maximum colony size was the same at both locations. Caution is required about these results because they are based on retrospective analysis rather than an experimental design to formally test the effects of reef locations and habitat types.

Colony growth measured as maximum diameter appears to be constrained in Florida because only a small percentage of colonies grew larger than 50 cm maximum diameter by year four. This may represent a present-day functional size limit. This limit may incorporate the effects of fragmentation from wave action [91] and the balance between axial extension at the growing tips and mortality due to basal predation by the territorial damselfish *Stegastes planifrons* [92] or the coralivorous snail *Coralliophila abbreviata*. Growth could also slow at larger sizes as branching patterns change or when metabolic resources are redistributed for other processes, such as reproduction [85]. While most *A*. *cervicornis* colonies in the natural population are also relatively small, less than 50 cm maximum diameter in the Florida Keys [17, 18], a few significantly larger colonies exist in the Dry Tortugas [30, 93]. In Puerto Rico, growth results from a natural population that is recovering after five years of no bleaching or disease exceeded Florida by several times [94]. The Puerto Rico results are noteworthy because they reveal that natural remnant populations retain the potential to grow and recover at rates that are some of the highest recorded for the species.

The condition of corals also matters when assessing restoration goals and *A*. *cervicornis* outplant condition declined with time across all project start dates. The natural population in the Florida Keys exhibited similar decreasing condition with increasing size [18]. Decreasing coral condition over time is important because it has negative implications for population growth. For example, in Puerto Rico, the growth and survival of *A*. *cervicornis* colonies declined significantly when partial mortality exceeded 20% [95]. Most colonies outplanted in the projects reported here exceeded this threshold at three years and exceeded 50% by four years. Various factors cause significant partial mortality including disease, predation by corallivores such as the gastropod *Coralliophila abbreviata* and the polychaete *Hermodice carunctulata* [28, 96, 97], and activity by damselfishes, chiefly *Stegastes planifrons* [92, 98]. Identifying causes of mortality could be relevant to restoration success if they can be mitigated by interventions [99]. However, even when interventions are possible such as removing corallivores [100], they are labor-intensive, effective only in the short-term, and ecological complexity can make it difficult to ascribe success to a particular intervention. For example, damselfish cause significant partial mortality to larger colonies of *A*. *cervivornis*, but they can also reduce the impact of corallivores [98]. An important result of projects reported here and elsewhere [86] is that high short-term survivorship of outplanted colonies is achievable without follow-on interventions.

The implication from our findings that genetics did not have a significant impact on survival, condition, or growth may be related to the small number of genotypes used initially, the small sample sizes of cohorts to start, and the lack of experimental design that tested for genotype effects. All nursery-raised genotypes used in these studies were selected for outplanting based on exhibiting normal growth patterns and rates. In other Florida-based studies, significant genotype effects among nursery-raised corals were seen in common growth experiments with some genotypes growing ten times faster than others [85], and others showing higher survivorship and faster growth after a coral bleaching event [101]. Significant growth rate differences were also observed among genotypes maintained in a coral nursery but not after outplanting [102]. Although different rearing methods can produce different growth forms, they may not lead to differences in calcification rates [103]. While identifying significant growth differences among colonies is interesting, the long-term survival of outplanted corals on reefs will reflect trade-offs between growth and other factors such as reproductive output, calcification rates, and resilience to disease and bleaching. How genetic variation affects adaptation and phenotypic plasticity in corals is an emerging area of research, including a focus on allelic and haplotypic richness within and among populations [104–108]. Ultimately, the principles of conservation genetics should inform coral outplanting practices, which requires information about coral population genetics [107, 109–111].

A significant challenge for *A*. *cervicornis* restoration practitioners is how to address the inevitable population declines that are caused by multiple sources of mortality. One approach is based on the idea of assisted evolution, in which selective breeding of corals or other genetic enhancements are used to produce corals that can thrive against the increasing frequency and magnitude of stressors, especially those related to warming and bleaching [112, 113]. There are, however, important lessons from the terrestrial realm that suggest super-coral progress will take decades or longer and require unprecedented amounts of funding. For example, work to restore the American chestnut (*Castanea dentata*), after it was nearly wiped out by a still pervasive introduced disease, started approximately a hundred years ago. Hybridization, backcrossing, and transformation with a resistance-conferring transgene achieved some successes. However, tens of millions of additional dollars are needed [114]. Coral bleaching and disease each present their own set of challenges related to coral physiology, microbiome dynamics, and a changing physical and chemical environment that makes the super coral approach more complicated than chestnut blight. Research to achieve super corals based on assisted evolution will undoubtedly advance our understanding of coral biology. However, coral practitioners need to be pragmatic about shorter-term approaches that might work to restore or help *A*. *cervicornis* populations recover.

Another approach to address high mortality rates is to repeatedly outplant large numbers of nursery-raised colonies to maintain or increase population numbers until stressors are mitigated or until the outplanted populations expand by themselves. This approach provides a general operational definition of restoration and is one way to measure success, even though it’s open-ended in terms of time and effort, not quantitative in terms of population criteria, and far short of recovery to conditions defined by historical baselines. Indeed, this approach describes the long-term commitment to restoration by the Coral Restoration Foundation^™^ and other agencies such as NOAA [115]. In addition, *A*. *cervicornis* outplanting projects, as they are conventionally conducted, address a narrow definition of restoration that focuses on a single species rather than restoring the structure and function of the ecosystem to the state before declines occurred [48]. Fortunately, there is detailed information from the 1970s and 1980s (before widespread declines) about the distribution and abundance of *A*. *cervicornis* for multiple reefs in the Florida Keys [116–119] that define a restoration target for what recovery might look like in the absence of stressors and the significant mortality events that result [31].

Notably, *A*. *cervicornis* recovered naturally following major hurricane destruction in 1960 at Dry Rocks, a reef with prolific amounts of *A*. *cervicornis*, where damage was not apparent five years later [116]. When a second storm (Hurricane Betsy) hit the same reef in September 1965, by October 1967 damage was once again not noticeable to trained geologists who had long-term experience at the site [116, 120]. The absence of major regional and global stressors in the 1960s, and the rapid growth of *A*. *cervicornis*, clearly explains the quick recovery after the two storms. However, in the absence of stressor mitigation, specifically related to global warming, practical restoration objectives must acknowledge that full and rapid recovery to historical baselines is not a realistic goal. Indeed, the NRP for *A*. *palmata* and *A*. *cervicornis* recognizes the challenges of restoration conducted against a background of mounting stressors. As a result, the NRP notes that restoration of the species will take 400 years, mainly due to estimates of how long it will take to decrease stressors related to increased warming [121] and associated increased frequency and intensity of coral bleaching and disease [122–124].

Therefore, we suggest that a working definition of restoration success for *A*. *cervicornis* is to attain self-sustaining populations started from outplanted nursery-raised corals that spawn annually and that have demographic and genetic characteristics similar to the remnant natural populations of the region. This definition falls short of recovery to historical baselines as a restoration goal because it is unrealistic to expect that nursery-raised corals will perform better than the remnant populations from which they were collected [112, 125]. The NRP contains population-based criteria that can help refine the above definition of restoration success. For example, based on abundance, coral thickets must encompass approximately 5% of forereef habitats between 5 and 20 m depth throughout the range of the species, with thickets composed of colonies ≥ 50 cm maximum diameter at a density of 1 colony per m^2^, or live benthic cover of approximately 25%. Populations must also be maintained for 20 years with these characteristics, based on a balance between sexual and asexual recruitment to maintain existing genetic diversity. The 5% criterion was selected to represent “a small portion of potential core habitat strata with the assumption that, under this condition, additional, lower-density stands would occupy additional habitat strata [6].” While 5% seems like a reasonable goal, current coral outplanting capacity will need to be significantly scaled up, long-term survivorship needs to increase, or both, to approach this restoration target within a decadal time-frame for select reefs (Table 4). The NRP also acknowledges that to meet or exceed the 5% criterion at the regional scale stressor mitigation and natural recovery will be required.

If stressors are diminished to a point where natural recovery begins, defined by net population growth, then a simple calculation demonstrates what recovery might look like in the absence of significant mortality. For example, if an outplanted population of 1050 corals grows at a rate of 10% per year [126] and ignoring carrying capacity constraints, then after ten years the population would increase to 2723 colonies, based on the standard exponential growth equation. Without continual outplanting effort, a 10% growth rate does not lead to a particularly meaningful population increase considering the high mortality results presented here. On the other hand, if an outplanted population of 1050 corals nearly doubles per year with exponential population growth, it would reach 643,761 colonies in ten years. While doubling a population per year might appear unrealistic, Shinn calculated that a small *A*. *cervicornis* colony of ten branches could generate 59 km of branches in ten years in the absence of what he referred to as pruning [116]. The rapid recovery of *A*. *cervicornis* populations is therefore possible under ideal conditions, starting from low outplanting numbers. Recovery in two years after Hurricane Donna devastated *A*. *cervicornis* at Dry Rocks in 1960 [116] and current recovery after five years at several locations in Puerto Rico [94] supports the idea that population growth can be rapid when stressors are absent.

What then is needed to achieve NOAA Recovery Plan success criteria, other than stressor abatement? Innovations in outplanting technology, identifying sites that promote survival and growth, and increases in the number, survivorship, and condition of colonies outplanted are critical requirements. For example, large numbers of individual *A*. *cervicornis* could be strategically outplanted to locations where the conditions are known to promote survival and growth; these include conditions such as low algae cover [99] and high hydrodynamic flow [89, 90]. However, there are likely stochastic elements to survivorship from year-to-year and site-to-site that makes it challenging to identify the best sites to achieve high survivorship–or even if there are best sites. Frequent and detailed monitoring may identify causes of mortality that are specific to some reef sites and not others, such as disease or high predation. Until then, outplanting efforts should continue to focus on sites defined by the historical distribution of the species. Pilot studies may also be useful where smaller numbers of colonies are outplanted to evaluate habitat suitability year-to-year before numbers ramp up significantly.

w attachments techniques are also needed instead of the commonly used method that relies on cementing individual corals to the benthos, one-at-a-time. If new approaches to outplanting increase colony numbers by an order of magnitude, say to 10,000 colonies per outplanting event, 27,183 colonies would result after ten years with a 10% growth rate under logistic population growth. If survivorship after ten years is 15%, then the remaining 4077 colonies would restore approximately 1346 m^2^ of habitat (with one third reaching 50 cm or greater maximum diameter). If survivorship improved to 40%, then the remaining 10,873 colonies (with one third greater than 50 cm maximum diameter) would restore approximately 3588 m^2^ of habitat. For *A*. *cervicornis* at depths of 5–10 m, the projected area of 3588 m^2^ equates to 2.1%, 19%, and 54% of the historical distributions at Carysfort (3588 m^2^/166,876 m^2^), Molasses (3588 m^2^/19,393 m^2^), and Conch Reefs (3588 m^2^/6679 m^2^), respectively. Recovery to meet the NOAA abundance criterion for the two smallest reefs would thus be attainable at the scale of decades, but only if survivorship is 40%. These numbers reflect outplanting one cohort at the start of the decade. The restoration times would go down substantially if cohorts are outplanted year-after-year, if survivorship significantly increases, or both.

While the projected area of 3588 m^2^ does not meet the NRP criterion that populations maintain themselves for 20 years, it provides a rough estimate of what would be required to restore a population, based on thicket abundance and size under the NRP definition. It is important to note that there is ecological value in even partially restored populations based on reports of higher fish abundance than degraded populations [127–130]. Fish and mobile invertebrates were not assessed in our studies, but they could easily be included in future monitoring programs [99, 127]. It may also be pragmatic to regard lush, continuous cover by acroporid corals to be an exceptional and ephemeral phenomenon in the presence of existing stressors. Although highly desirable, thickets may grow or shrink over time [131]. Restored populations that are reduced in abundance and size, even substantially below NRP metrics, still might function as reserve or seed populations that simply persist until favorable conditions allow for growth. The number of enduring reserve populations at multiple locations might be considered another measure of restoration success.

In addition to innovations in outplanting technology to increase numbers and survivorship, new monitoring techniques will be needed to replace the in situ counting and measurement of individual corals conducted to date. In particular, high-definition photo-mosaics [132–134] combined with computer-enhanced point count metrics [135] can be used to measure the performance of thousands of outplanted corals, addressing NRP abundance and size metrics that describe the status of populations.

Our findings suggest that restoration projects have an important role to play in the persistence and recovery of *A*. *cervicornis*. The NRP specifically calls for “active population enhancement” using offshore nurseries and other strategies to improve population densities and genetic diversity. However, many constraints related to historical conditions and existing stressors remain [136, 137]. Indeed, as early as 1964 it was recognized that reduced reef growth occurs opposite large tidal passes in the Florida Keys [138]. At least half of all potential reef tract area in the Florida Keys was also not considered suitable for long-term coral growth [116]. Long-term commitments to outplanting and monitoring will be needed to identify habitat types, reef locations, and ecosystem interactions that impact survival rates. In addition, modeling efforts can help forecast the trajectories of coral populations [139]. Although the data with which to parameterize such models are limited [126, 140–143], our results provide a start. Given the fast growth rate of *A*. *cervicornis* observed in this study and elsewhere [4, 60, 65, 94], recovery of the species could be rapid under suitable conditions [31, 112, 116] and if the adaptive potential of populations is maximized [144]. However, until suitable conditions arise, the capacity to grow large numbers of healthy colonies in offshore nurseries and the increasing capacity of restoration programs to outplant large numbers of genetically diverse colonies provides *A*. *cervicornis* with important protection against local extinction. This approach also applies to other coral species listed as Threatened under the Endangered Species Act. In particular, its congener *A*. *palmata* and the large mounding corals *Orbicella faveolata* and *O*. *franksi*, which are the focus of emerging technologies [145] to grow in nurseries for outplanting to offshore reefs in Florida.

## Supporting information

S1 Table Total areas (m^2^) by habitat type and depth for sanctuary preservation areas located at Carysfort reef, molasses reef, and conch reef. Check marks indicate habitats used for two depth ranges to evaluate time and effort required to meet recovery metrics identified in the NOAA Recovery Plan. Suitable habitat was determined based on historical distribution of *Acropora cervicornis* in Florida.
(DOCX)
