# Supplementary material for: Survivorship and growth in staghorn coral (Acropora cervicornis) outplanting projects in the Florida Keys National Marine Sanctuary
Source: PLoS One. 2020 May 6;15(5):e0231817. doi: 10.1371/journal.pone.0231817 (PMC7202597; doi:10.1371/journal.pone.0231817)
Supplement: S1 Table — Check marks indicate habitats used for two depth ranges to evaluate time and effort required to meet recovery metrics identified in the NOAA Recovery Plan. Suitable habitat was determined based on historical distribution of Acropora cervicornis in Florida. (DOCX) [file pone.0231817.s001.docx]

| **Carsyfort Reef Habitat** | **Depth (m)** | **Suitable** | **Area (m^2^)** | **5% Area (m^2^)** |
| --- | --- | --- | --- | --- |
| High-relief spur & groove | <5 |  | 230,000 |  |
| High-relief spur & groove | 5–10 | √ | 799,437 | 39,972 |
| High-relief spur & groove | 10–20 | √ | 618,378 | 30,919 |
| High-relief spur & groove | >20 |  | 69,258 |  |
| Low-relief spur & groove | <5 |  | 170,000 |  |
| Low-relief spur & groove | 5–10 | √ | 511,289 | 25,564 |
| Low-relief spur & groove | 10–20 | √ | 808,727 | 40,436 |
| Low-relief spur & groove | >20 |  | 111,965 |  |
| Medium-relief patch-reef | <5 |  | 20,000 |  |
| Medium-relief patch-reef | 5–10 | √ | 140,400 | 7,020 |
| Continuous low-relief hardbottom | <5 |  | 70,000 |  |
| Continuous low-relief hardbottom | 5–10 | √ | 1,824,373 | 91,219 |
| Continuous low-relief hardbottom | 10–20 | √ | 88,870 | 4,443 |
| Continuous low-relief hardbottom | >20 |  | 30,593 |  |
| Patchy low-relief hardbottom | 5–10 | √ | 62,025 | 3,101 |
| Patchy low-relief hardbottom | 10–20 | √ | 60,000 | 3,000 |
| Patchy low-relief hardbottom | >20 |  | 10,000 |  |
| Rubble | <5 |  | 10,000 |  |
| Sand | 10–20 |  | 14,990 |  |
| Seagrass | 5–10 |  | 37,999 |  |
| All suitable habitat | 5–10 |  | 3,337,524 | 166,876 |
| All suitable habitat | 10–20 |  | 1,575,975 | 78,799 |
| All suitable habitat | 5–20 |  | 4,913,499 | 245,675 |

| **Molasses Reef Habitat** | **Depth (m)** | **Suitable** | **Area (m^2^)** | **5% Area (m^2^)** |
| --- | --- | --- | --- | --- |
| High-relief spur & groove | <5 |  | 60,000 |  |
| High-relief spur & groove | 5–10 | √ | 153,186 | 7,659 |
| High-relief spur & groove | 10–20 | √ | 128,250 | 6,413 |
| High-relief spur & groove | >20 |  | 13,827 |  |
| Low-relief spur & groove | <5 |  | 30,000 |  |
| Low-relief spur & groove | 5–10 | √ | 91,053 | 4,553 |
| Low-relief spur & groove | 10–20 | √ | 45,157 | 2,258 |
| Low-relief spur & groove | >20 |  | 26,413 |  |
| Medium-relief patch-reef | <5 |  | 8,054 |  |
| Continuous low-relief hardbottom | <5 |  | 50,038 |  |
| Continuous low-relief hardbottom | 5–10 | √ | 143,619 | 7,181 |
| Continuous low-relief hardbottom | 10–20 | √ | 19,855 | 993 |
| Patchy low-relief hardbottom | <5 |  | 50,000 |  |
| Rubble | <5 |  | 195,389 |  |
| Rubble | 5–10 |  | 10,000 |  |
| Seagrass | <5 |  | 70,120 |  |
| Seagrass | 5–10 |  | 1,025 |  |
| All suitable habitat | 5–10 |  | 387,859 | 19,393 |
| All suitable habitat | 10–20 |  | 193,262 | 9,663 |
| All suitable habitat | 5–20 |  | 581,121 | 29,056 |

| **Conch Reef Habitat** | **Depth (m)** | **Suitable** | **Area (m^2^)** | **5% Area (m^2^)** |
| --- | --- | --- | --- | --- |
| Continuous low-relief hardbottom | <5 |  | 11,874 |  |
| Continuous low-relief hardbottom | 5–10 | √ | 58,615 | 2,931 |
| Continuous low-relief hardbottom | 10–20 | √ | 150,715 | 7,536 |
| Continuous low-relief hardbottom | >20 |  | 16,249 |  |
| Low-relief spur & groove | 5–10 | √ | 74,963 | 3,748 |
| Low-relief spur & groove | 10–20 | √ | 234,415 | 11,721 |
| Low-relief spur & groove | >20 |  | 300,875 |  |
| Rubble | <5 |  | 19,622 |  |
| Undefined | >20 |  | 7,346 |  |
| All suitable habitat | 5–10 |  | 133,578 | 6,679 |
| All suitable habitat | 10–20 |  | 385,130 | 19,256 |
| All suitable habitat | 5–20 |  | 518,707 | 25,935 |
